# The Reliability and Validity of Liu´s Self-Report Questionnaire for Screening Putative Pre-Psychotic States (BQSPS) in Adolescents

**DOI:** 10.1371/journal.pone.0167982

**Published:** 2016-12-14

**Authors:** D. Núñez, V. B. Arias, S. Campos

**Affiliations:** Faculty of Psychology, Universidad de Talca, Talca, Chile; University of California Los Angeles, UNITED STATES

## Abstract

The usage of rigorous analyses based on contemporary methods to enhance psychometric properties of screening questionnaires aimed to address psychotic-like experiences (PLE) is currently being encouraged. The Brief Self-Report Questionnaire for Screening Putative Pre-psychotic States (BQSPS) is a recently created tool addressing PLE beyond attenuated positive symptoms (APS). Its psychometric properties as a screening tool for first step assessment seems to be adequate, but further research is needed to evaluate certain validity aspects, particularly its dimensionality, internal structure, and psychometric properties in different populations. We assessed the reliability, construct validity, and criterion validity of BQSPS in two samples: 727 adolescents aged 13–18 years, and 245 young adults aged 18–33 years. We used exploratory structural equation modeling (ESEM), confirmatory factor analysis (CFA), and Structural Equation Modeling (SEM). The original four-factor structure was not replicated. The best fit in adolescents was obtained by a structure of three-correlated factors: social anxiety (SA), negative symptoms (NS), and positive symptoms (PS). This structure was confirmed in young adult subjects. The three-factor model reached a predictive capability with suicidality as external criterion. PLE are represented by a three-factor structure, which is highly stable between adolescent and young-adult samples. Although the BQSPS seems to be a valid tool for screening PLE, its psychometric properties should be improved to obtain a more accurate measurement.

## Introduction

Developing strategies aimed at identifying individuals at high clinical risk of first episode psychosis is one of the major current goals of psychiatric services worldwide [1]. These strategies are increasingly focused on the early detection of subjects showing subthreshold symptoms comprising positive and negative symptoms, and functional difficulties developed in the period preceding the onset of psychosis [2]. The role of these attenuated psychotic symptoms–also termed psychotic-like experiences (PLEs)–as specific predictors of psychosis remains unclear [3,4]. However, cumulative evidence shows associations between persisting PLEs and suicidality [5], higher risk of psychiatric disorders [6], and functional impairments in ultra-high- risk (UHR) populations [7,8], general adolescent populations [9], and help-seeking adolescent subjects [10].

Operational criteria and different measures aimed to enhance the early detection of people at risk of psychosis have been created and tested during the last two decades. Daneault et al. [11] identified 22 instruments with the most widely used measures being clinician administered interviews addressing previously well-validated clinical high-risk criteria [12]. The prognostic accuracy of these thorough psychometric measures is sufficient for help-seeking subjects with psychiatric symptoms [2]. But because the usage of these instruments is unsuitable either in the general population [13,14] or in primary health care settings, in recent years, several brief, easy-to-use self-report questionnaires have proliferated for screening purposes [14]. Under the general framework of the clinical high-risk approach [15], these tools have been mainly focused on predicting the transition to psychosis rather than in construct specificity per se [16].

Despite the relatively expanded usage of these screening questionnaires, different issues have recently been highlighted, that prevent against a clear recommendation of screening measures for PLE for psychosis. For instance, studies analyzing their psychometric properties, beyond sensitivity and specificity, are surprisingly scant. In fact, a recent review identified 17 screening tools, and evidence about validity and reliability was found for very few of them [13]. In addition, knowledge on the structure of PLEs and how they can accurately be captured by the available brief self-administered questionnaires is still inconclusive. The Community Assessment of Psychic Experiences (CAPE-42) [17,18] and the shorter version, CAPE-20 [7,19, 20], likely contain the most cumulative evidence about PLE factor structure, showing a similar and stable structure with three to five factors. Although some initial evidence has been reported for the new brief version (CAPE-P15) [21], the controversy remains, and mirrors the current theoretical debate about the structure of the psychopathology, organized around two main perspectives: the multidimensional [7,18,22] and the unidimensional approaches [23,24]. Both perspectives are supported by recent studies using advanced statistical techniques. Whereas the former was supported by Therman and Ziermans [25] who confirmed the three-factor structure (persecutory ideation, bizarre experiences, and perceptual abnormalities), the latter was supported by Núñez et al. [26], who found that the underlying structure of the CAPE-P15 can be adequately represented by a general factor and three separable specific traits. This suggests that there might be a common source underlying the subclinical psychotic symptoms addressed by the scale.

The need for developing shorter questionnaires with robust psychometric properties is currently well recognized [11, 13, 16]. Consequently, the usage of rigorous analyses based on contemporary methods is strongly encouraged nowadays. Recently, a self-report screening for pre-psychotic putative symptoms (BQSPS) [27] was created. Unlike other questionnaires developed to improve the predictive validity regarding the transition to psychosis, the BQSPS aims to detect early and broadly at risk mental states characterized by subtle symptoms and functional impairments [28]. Additionally, the BQSPS not only addresses attenuated positive symptoms, like most of the available screening questionnaires [16], but also includes other subthreshold clinical manifestations. This fits with evidence showing that APS are recognized as part of a late and severe stage in the development of psychotic disorders [29], and that they have not been entirely useful to predict the transition to psychosis [4, 30–32]. Moreover, it is in line with findings demonstrating that the concurrence of both positive and negative symptoms increases the risk for schizophrenia [33]. The BQSPS is a 15-item scale with four categories: interpersonal difficulty/social anxiety symptoms, self-deprecating descriptions, negative symptoms, and subthreshold psychotic-like experiences. According to the author’s analyses, the BQSPS has certain construct validity [27], and it can be useful to reliably distinguish clinical from non-clinical samples. A recent study found a moderate to large convergent validity, acceptable internal consistency for each scale, and modest test–retest reliability, recommending its usage for screening PLEs in college populations [34]. Nevertheless, and critically for the present study, factor analyses demonstrating the accuracy with which the proposed four-category model represents the data have not yet been reported. Consequently, in order to be truly useful for further investigation, and to perform screening for first step risk assessment, its internal structure must be thoughtfully tested, in a more heterogeneous sample of adolescents, arguably the most identifiable at-risk population [35].

We used exploratory and confirmatory analyses to examine the internal structure of the BQSPS. Moreover, we attempted to expand the scope of the research on and use of the BQSPS from the population studied by Liu et al. (a Chinese-speaking, relatively small sample of subjects with different at-risk levels) to a broader population of Spanish-speaking adolescents aged 13–18. Finally, based on evidence demonstrating associations between subthreshold psychotic symptoms and suicidality [36], and revealing that the former may be reliable indicators of the latter in both adolescent and adult general populations [37, 38], we tested the differential relationships between suicidality and BQSPS factors as predictive latent variables.

## Material and Methods

### 2.1 Participants

This cross-sectional study was conducted with 727 high school students (adolescents) (women = 50.7%) aged 13–18 years (mean = 15.4 ± 1.33), recruited between April and July 2014 in six secondary schools of the city of Talca, Chile. All students between the ages of 13–18 years who were formally registered in school were invited to participate. Only two subjects declined to participate in the study, and thus, nearly 100% of the subjects attending each school were recruited. We observed that 5.9% of subjects had one or more missing values, with a distribution completely at random (MCAR, Little's test sig. = 0.142). Thus, cases with missing values were dropped out. We conducted the CFA analyses with a final sample of 684 subjects (see S1 File), and the SEM analyses with a final sample of 669 (see S2 File) subjects. To provide evidence about the generalizability of the results, we used a sample of 245 university students (young adults) (women = 75.4%) aged 18–33 years (mean = 20.4 ± 2.5). The only inclusion criterion was that the students voluntarily agreed to participate in the study. Only one subject declined to participate in the study. No missing data were observed in the university student sample.

### 2.2 Measures

The BQSPS [27] is a 15-item self-report questionnaire aimed to capture the early and broadly defined at risk mental status. It addresses four symptomatic categories (interpersonal difficulty/social anxiety symptoms, self-deprecating descriptions, negative symptoms, and subthreshold psychotic-like experiences). Responses to items ranged from 1 (never) to 5 (very often). Additionally, we addressed suicidality by the Okasha Suicidality Scale [39, 40]. It is a self-administered screening questionnaire with four items. Items 1, 2 and 3, address suicidal ideation, and item 4 addresses suicide attempt. In the current version, the items ranged from 1 (never) to 5 (very often).

### 2.3 Procedure

We translated and adapted the English version of the questionnaire to the Spanish language [41, 42] (see S1 Table).

We conducted the study in those public schools who agreed to participate after meetings with directive committees. Researchers participated in different parent’s meetings to present the research project. We also conducted the study in the Faculty of Psychology of the Universidad de Talca. We explained the project to both the directive committee and students. After its approval and once written informed consents were obtained from both the caregivers of adolescents and young adults themselves, the participants completed the questionnaires, administered in a classroom setting by trained psychologists.

Ethical approval was obtained from the Bioethics Committee of Universidad de Talca.

### 2.4 Statistical analysis

First, we explored the scale structure through exploratory structural equation models (ESEM) [43] with geomin oblique rotation. Based on the unidimensional solution, we estimated different models adding a latent variable in each iteration (models M1-M4). In order to find a balance between fit indices, parsimony and interpretability of the factor-loading pattern, we selected the three-factor solution, which was confirmed by confirmatory factor analysis (CFA; model M5). We additionally estimated with CFA the four-factor model (M6) proposed by Liu et al. To provide evidence about the generalizability of the results obtained in adolescents, we tested both the three and four factor confirmatory models in an independent sample composed by college students. Finally, we estimated a structural equation model with BQSPS factors as predictors, and suicidality as criterion (model M9).

Given the ordinal nature of our data, we used the Weighted Least Squares Means and Variance-adjusted estimation method, performed through MPlus v. 7.3 [43].

## Results

The fit indices of tested models are shown in Table 1. Regarding the ESEM models, the RMSEA of M4 model did not substantially improve relative to the more parsimonious M3 model, with three-correlated factors: social anxiety (SA), negative symptoms (NS) and positive symptoms (PS).

**Table 1:** Fit indices of the CFA, ESEM and SEM models.

Model	Type	Factors	Sample	RMSEA	CFI	TLI	Chi-sq	DF	FP
M1	ESEM	1	Adolescents	0.086	0.862	0.839	545.11	90	75
M2	ESEM	2	Adolescents	0.066	0.932	0.906	299.45	76	89
M3	ESEM	3	Adolescents	0.044	0.975	0.958	145.09	63	102
M4	ESEM	4	Adolescents	0.038	0.985	0.969	100.14	51	114
M5	CFA	3	Adolescents	0.064	0.927	0.912	327.84	87	78
M6**	CFA	4	Adolescents	0.078	0.893	0.866	436.95	84	81
M7	CFA	3	Adults	0.055	0.963	0.956	153.31	87	78
M8**	CFA	4	Adults	0.084	0.917	0.896	234.68	84	81
M9	SEM	3	Adolescents	0.052	0.976	0.972	460.32	164	106

Note. ESEM = Exploratory structural equation model; CFA = Confirmatory factor analysis; SEM = Structural equation model; Factors: Number of factors specified by the model; RMSEA = Root mean square error of approximation; CFI = Comparative fit index; TLI = Tucker-Lewis index; Chi-sq = Chi square value; DF = Degrees of freedom; FP = Free parameters.

**The residual covariance matrix is not positive definite.

Moreover, the load factor structure of M4 was not clearly interpretable (the loading configuration of factor 4 was not interpretable; see Table 2), unlike the better defined load pattern reached by M3. In this latter model, the items were grouped in three correlated clusters with content similarities: social anxiety (SA, 7 items), negative symptoms (NS, 4 items), and positive symptoms (PS, 4 items). The cross-loadings of ME were low, except for items 3, 4, and 13. A new estimation of M3 by CFA (M5) revealed an acceptable fit (RMSEA = .064; CFI = .927; TLI = .912), substantially better than the theoretical model proposed by Liu et al. (M6; ΔRMSEA = -0.014, ΔCFI = 0.034, ΔTLI = 0.046) [44, 45]. We replicated these results in the adult sample, where the fit of the confirmatory model of three factors (M7) was substantially better than the fit of the theoretical four-factor model (M8, see Table 1). Factor loads of M7 are depicted in S2 Table.

**Table 2:** Factor loadings of the three and four factor ESEM models (adolescent sample).

Model	ESEM four factors (M4)				ESEM three factors (M3)		
Item/factor	F1	F2	F3	F4	F1	F2	F3
1	0.712*	0.200*	0.085	0.037	0.681*	-0.151	0.087
2	0.696*	-0.009	0.042	-0.047	0.700*	0.008	0.028
3	0.458*	0.294*	0,117	-0.034	0.446*	0.277*	0.116
4	0.010	0.481*	0.248*	-0.039	0.008	0.386*	0.263*
5	0.554*	-0.020	-0.021	-0.335*	0.646*	-0.095	-0.057
6	0.131	0.082	0.126	0.061	0.102	0.078	0.144
7	0.516*	0.079	0.010	-0.056	0.524*	0.088	-0.007
8	0.604*	0.006	-0.069	-0.189*	0.657*	-0.006	-0.105
9	0.115	0.593*	-0.006	0.087	0.051	0.635*	-0.005
10	-0.019	0.562*	-0.017	0.288*	-0.147	0.657*	0.008
11	0.621*	0.093	-0.097	0.079	0.566*	0.195*	-0.106
12	0.564*	0.005	0.012	0.333*	0.410*	0.161*	0.043
13	0.273*	0.096	0.343*	0.025	0.256*	0.025	0.376*
14	0.007	-0.050	0.861*	0.049	0.000	-0.273	0.950*
15	0.003	0.247*	0.450*	-0.235*	0.099	0.030	0.441*

Note:ESEM = Exploratory structural equation model

* = significant (*p* < .01)

**Fig 1:**
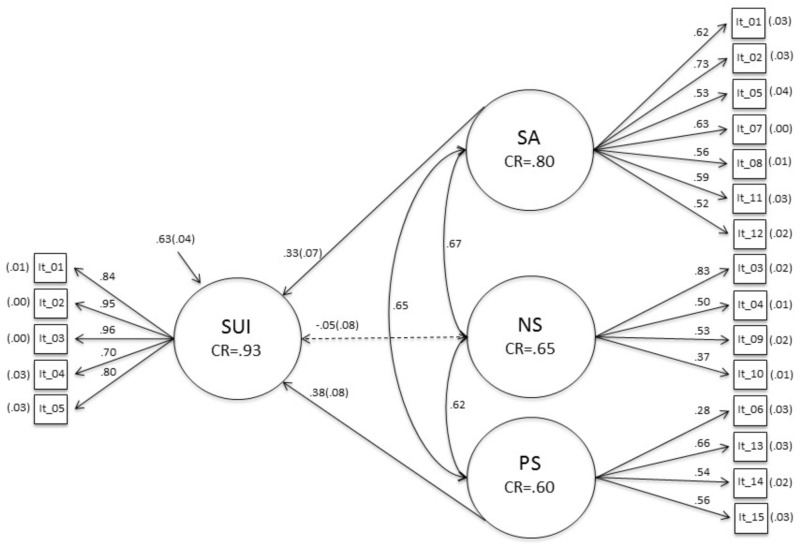
Parameters of model M9. Standard errors are in parentheses. SA = Social anxiety; NS = Negative symptoms; PS = Positive symptoms. SUI = Suicidal ideation; CR = Composite reliability.

Between-factor correlations in M5 were moderate (Fig 1). Congruence coefficients (Cr) between factor loads of M5 and M7 were high (.99, .97, and .95) [46]. The composite reliability (Cr) of SA was sufficient, but moderately low for PS and NS (SA-Cr = .80, NS-Cr = .65, PS-Cr = .60). Finally, the fit of the structural model (M9) model was good (RMSEA = .052; CFI = .976; TLI = .972). The regression path from PS toward suicide ideation was significant and low (.38). A similar result was observed for SA (.33). NS showed a non-significant negative regression path (-.05).

## Discussion

We examined the internal structure of early and broadly at risk mental states characterized by subtle psychotic symptoms addressed by the Brief Self-Report Questionnaire for Pre-psychotic Putative Symptoms (BQSPS) [27] in a sample of non-help-seeking adolescents aged 13–18 years. Our results did not replicate the original four-factor structure. The best fit and interpretability was obtained by a structure of three -correlated factors: social anxiety (SA), negative symptoms (NS), and positive symptoms (PS). This model was confirmed in an independent sample of young adults. SA comprises seven items addressing aspects related to anxiety about social interaction, feelings of being emotionally distant, and having few social skills. NS addresses four items referring to feelings of tiredness and lethargy and concentration difficulties. PS involves four items associated to perceptual anomalies and paranoid ideation.

We used contemporary methods suitable for analyzing some critical issues about the early detection of subthreshold psychotic symptoms,—for instance, the structure of these pre-clinical manifestations in the general population [25]. According to our knowledge, this is the first study specifically aimed at testing the structure of early and broad at risk mental states addressed by the BQSPS. Our result showing three factors distinct enough from each other fits in with the multidimensional approach according to which subthreshold psychotic symptoms should not be regarded as a homogeneous entity [47, 48].

Based on this finding, and given the adequate construct validity of the BQSPS, it could be used as a multidimensional screening for sub-psychotic experiences, in the context of new approaches not only aimed to prevent psychosis, but also other psychiatric disorders [29]. Nevertheless, there are some issues requiring clarification by further research before a clear recommendation for it usage.

First, our finding revealed a better goodness of fit for the three-factor structure relative to the four-factor structure originally proposed [27]. When accounting for these structural differences, some relevant aspects deserve mentioning. We observed that the items comprising the prior factor termed “self-deprecating descriptions” (1, 4 and 9) were subsumed in both SA (item 1) and NS factors (items 4 and 9). Moreover, SA included two additional items previously defined as negative symptoms (items 3 and 11). As outlined in Table 2, the former (“I feel lethargic whatever I do”) presents an unclear and theoretically inconsistent loading pattern (i.e., higher loading scores for the SA factor) either in the four-factor model or in the three-factor model, possibly because the item wording is confusing for adolescents. Therefore, it should be modified or excluded in future research. Additionally, our NS factor acquired a new structure (the two self-deprecation items 4 and 9, plus the original item 10). Finally, the most stable items were those addressing attenuated positive symptoms, all of them remaining as originally proposed.

Second, despite the adequate construct validity of the scale, a higher accuracy could be obtained if some items were slightly modified. Particularly, the moderately low reliability and variance explained by PS subscale could be improved by re-wording some items. For instance, it is reasonable to think that “being worried about loyalty of friends” might be an item confounding paranoid ideation with certain normal reactions of adolescence. This is supported by our results revealing a good functioning of this item in adults (λ = .609) but not in adolescents (λ = .28).

The three-factor model reached its own explicative capability on suicidality as criterion. SA and PS showed low and positive significant correlations with this criterion. The explanative contribution of SA was incremental with respect to PS, which means that including social anxiety indicators contribute to predicting suicidality beyond the generic psychotic-like symptoms. Overall, recent research examining the relationships between suicidality and psychiatric symptoms suggests the existence of differential and specific associations [49–52]. Concerning psychotic risk symptoms, Granö et al. [53] found that visual distortions explained suicidal ideation when other psychotic risk symptoms and demographic variables were controlled in a sample of help-seeking adolescents. Additionally, Fujita et al. [54] observed that auditory verbal hallucinations increase the risk for suicide attempts in a clinical sample of adolescents with suicidal ideation. The knowledge about the nature of this relation in non-clinical samples is scarce and contradictory [55]. Alternately, Koyanagi et al. [56] recently found that each psychotic experience, regardless of the type, was independently associated with suicide ideation in adults. In contrast, DeVylder and Hilimire [57] reported specific associations between auditory hallucinations and suicidal ideation in young adult subjects, and Capra et al. [58] observed that perceptual abnormalities and persecutory ideation, but not bizarre experiences were specifically associated with an increased risk for suicide in young adults. Finally, Kelleher et al. [59] found a specific association between auditory hallucinations and higher rates of suicide attempt in adolescents. Because of the usage of different domains (mainly positive-like symptoms in the case of these prior investigations, and a broader pool of subthreshold symptoms beyond PS in our research), direct comparisons should be made with caution. Nevertheless, our results showing that both PS and SA might contribute to predict suicidality, support the existence of specific patterns of relationships between subtle psychotic experiences and suicidality in adolescents. This finding should be cautiously interpreted. Given the evidence showing that PLEs can represent a severity index of non-psychotic psychopathology [32,60], relationships between suicidality and PLE could merely reflect a higher underlying risk of suicidality as a function of higher severity of psychiatric symptoms or more severe levels of mental distress. Further research with broader samples of adolescents is necessary for a better understanding of these differential relationships, probably influenced by other mediating variables [38] or explained by shared risk factors as suggested by DeVylder et al. [55].

Given some existing controversy about screening PLE in community settings [13], our results highlight the importance of properly adapting measurement instruments to different populations according to their own characteristics, and fits in with recent literature encouraging increased focus on psychometric properties of questionnaires addressing psychotic experiences [13, 61]. We think that providing accurate evidence about psychometric properties of questionnaires addressing PLE may help researchers avoid risks associated with their usage in different cultural contexts.

In summary, a three-factor model can represent PLE addressed by the BQSPS. This model was highly stable between adolescent and adult samples. Although the BQSPS seems to be a valid tool for screening PLEs, it could be improved by either rewording some items or testing new items with better psychometric properties. Additionally, the three-factor model of PLE reached certain explicative capabilities on suicidality; SA and PS being the factors with higher correlations with this criterion.

Some limitations deserve mention. First, we did not address clinical samples. To investigate the functioning of the measurement in diagnosed individuals could provide new insights about the PLE. Second, we did not use a random sampling method. Although the distribution of the age showed an equiprobable distribution in both male and female adolescents, a random sampling would be helpful to reduce a possible sampling bias. Finally, because of our cross-sectional design, causal relationships cannot be inferred from the present findings.

## Supporting Information

S1 File CFA Data Set. (XLSX)

S2 File SEM Data Set. (XLSX)

S1 Table Adapted items in Spanish. (DOCX)

S2 Table Factor loadings of the three-factor CFA model (adult sample). (DOCX)
