# Supplementary material for: The Reliability and Validity of Liu´s Self-Report Questionnaire for Screening Putative Pre-Psychotic States (BQSPS) in Adolescents
Source: PLoS One. 2016 Dec 14;11(12):e0167982. doi: 10.1371/journal.pone.0167982 (PMC5156383; doi:10.1371/journal.pone.0167982)
Supplement: S1 Table — (DOCX) [file pone.0167982.s003.docx]

**S1 Table**: **Adapted Items in Spanish**

| Item | Content |
| --- | --- |
| 1 | No puedo enfrentar la presión asociada a las multitudes |
| 2 | Siento que no puedo sentirme cercano a la gente |
| 3 | Me siento lento con casi cualquier cosa que haga |
| 4 | Cuando leo o pienso, me fatigo fácilmente |
| 5 | Estoy casi siempre callado cuando estoy con otros |
| 6 | Me preocupo por la lealtad de mis amigos o colegas (compañeros) |
| 7 | Tiendo a guardarme mis sentimientos |
| 8 | Me siento nervioso cuando estoy hablando frente a un grupo grande de personas |
| 9 | Me es muy difícil concentrarme en las tareas |
| 10 | Casi todo lo que hago termina en desorden |
| 11 | No tengo una forma expresiva y animada de hablar |
| 12 | Soy malo para corresponder cortesías y gestos sociales |
| 13 | ¿Cuando ves personas hablando entre sí, a menudo te preguntas si están hablando de ti? |
| 14 | ¿Encuentras significados ocultos en las palabras o acciones de otros? |
| 15 | ¿Escuchas sonidos o voces que mencionan tu nombre cuando no hay nadie a tu alrededor? |
