# Supplementary material for: The Reliability and Validity of Liu´s Self-Report Questionnaire for Screening Putative Pre-Psychotic States (BQSPS) in Adolescents
Source: PLoS One. 2016 Dec 14;11(12):e0167982. doi: 10.1371/journal.pone.0167982 (PMC5156383; doi:10.1371/journal.pone.0167982)
Supplement: S2 Table — (DOCX) [file pone.0167982.s004.docx]

**S2 Table: Factor loadings of the three - factor CFA model (adult sample)**

| **Item/Factor** | SA | NS | PS |
| --- | --- | --- | --- |
| 1 | 0,691 |  |  |
| 2 | 0,797 |  |  |
| 5 | 0,703 |  |  |
| 7 | 0,668 |  |  |
| 8 | 0,585 |  |  |
| 11 | 0,659 |  |  |
| 12 | 0,646 |  |  |
| 3 |  | 0,747 |  |
| 4 |  | 0,620 |  |
| 9 |  | 0,764 |  |
| 10 |  | 0,609 |  |
| 6 |  |  | 0,609 |
| 13 |  |  | 0,664 |
| 14 |  |  | 0,614 |
| 15 |  |  | 0,340 |
| CR | 0,85 | 0,78 | 0,64 |
| Note: SA = Social Anxiety; NS = Negative Symptoms; PS = Positive Symptoms; CR = Composite reliability | | | |
|  |  |  |  |
